# Affect, Belief, and the Arts

**DOI:** 10.3389/fpsyg.2021.757234

**Published:** 2021-12-02

**Authors:** Rami Gabriel

**Affiliations:** Research Group in Mind, Science, and Culture, Department of History, Humanities, and Social Sciences, Columbia College Chicago, Chicago, IL, United States

**Keywords:** affect, ecological psychology, salience, extended evolutionary synthesis, affective neuroscience, mimesis, ritual, immanence

## Abstract

The cultural project is a therapeutic melding of emotion, symbols, and knowledge. In this paper, I describe how spiritual emotions engendered through encounters in imaginative culture enable fixation of metaphysical beliefs. Evolved affective systems are domesticated through the social practices of imaginative culture so as to adapt people to live in culturally defined cooperative groups. Conditioning, as well as tertiary-level cognitive capacities such as symbols and language are enlisted to bond groups through the imaginative formats of myth and participatory ritual. These cultural materializations can be shared by communities both synchronically and diachronically in works of art. Art is thus a form of self-knowledge that equips us with a motivated understanding of ourselves in the world. In the sacred state produced through the arts and in religious acts, the sense of meaning becomes noetically distinct because affect infuses the experience of immanence, and one's memory of it, with salience. The quality imbued thereby makes humans attentive to subtle signs and broad “truths.” Saturated by emotions and the experience of alterity in the immanent encounter of imaginative culture, information made salient in the sacred experience can become the basis for belief fixation. Using examples drawn from mimetic arts and arts of immanence, I put forward a theory about how sensible affective knowledge is mediated through affective systems, direct perception, and the imagination.

## Introduction

In this paper, I explore the relationship between belief, art, and religion through describing sensible affective knowledge in imaginative culture^1^. The foundational insight I aim to articulate is that belief fixation arises through an assemblage of emotion, symbols, and knowledge, which are delivered through ritual participation and art practices.

I argue that emotions evoked through ritual in art and religion play a significant role in belief fixation as part of the cultural evolution of imaginative culture. This is because participatory rituals compel feelings of immanence, which indicate heightened significance, or salience (section Spiritual Emotions). This spiritual emotion composed of awe, wonder, and transcendence is the indwelling, inherent, the plenitude within^2^. Philosophers have discussed this aspect of human existence as Dasein, presence, the noumenal, oceanic state, or the sublime. I take these to be contextualized versions of universal human emotions founded on the basic affective systems of PLAY, CARE, FEAR, and GRIEF that are then conditioned and associated into epistemic cultural niches^3^. I expand on how this numinous emotion is central to the maintenance of shared social norms as expressed through rituals that perpetuate the social order of the logos (section Ritual and Logos). As aspects of the epistemic niche, Art and the Humanities contain practices that enable modes of reflection and expressive formats for coping and knowledge (section Art as Self-Knowledge). Thus, drawing from insights in affective neuroscience and ecological psychology (section Emotions and Direct Perception), a theory of sensible affective knowledge as an embodied, imaginative set of reflexive practices is provided (section Sensible Affective Forms of Knowledge). I illustrate how emotions and reflexivity provide the means for belief fixation and sensible affective knowledge by exploring the mimetic arts (section Drama and Literature) and arts of immanence (section Arts of Immanence).

## Cultural Evolution

### Spiritual Emotions

We occupy an ecological niche of symbiotic links between place, geography, alimentary sources, weather, and other elements of the environmental landscape. Settled human habitation materializes our activities and conceptual categories in the way it is organized around ecological features like water sources, trees, and geological features. Our ancestors built infrastructure that now constitutes an extended ecological setting (Laland et al., 2015). Extended ecology includes man-made elements that solicit and direct thought. Think of how the layout of a classroom or a train station platform solicit particular behaviors and modes of attention. The cooperative activities that our species engage in are shaped by the extended ecological niche of cultural lifeways (Donati, 2010; Sterelny et al., 2013).

We inhabit an epistemic niche, called culture, that structures the ways we think and navigate social settings. Cultural artifacts like names, totems, techniques, social norms, hierarchies, and ontologies are traditions passed down through generations. They constitute a symbolic language that through associations and conditioning motivate our affective experiences. According to Roy Rappaport: “*Humanity is a species that lives and can only live in terms of meanings it itself must invent. These meanings and understandings not only reflect or approximate an independently existing world but participate in its very construction. The worlds in which humans live are not fully constituted by tectonic, meteorological and organic processes…but are also constructed out of symbolically conceived and performatively established cosmologies, institutions, rules, and values*” (Rappaport, 1999, p. 8)^4^.

Cultural establishment of conventions which shape the way individuals approach the world are reified, modified through language, and made into real things by social actions, like rituals (Rappaport, 1999, p. 9). They have a significant effect upon how individuals structure their goals, fears, and aspirations. One important cultural convention of norms and metaphysics is religion, it is a system that establishes the Truth upon which other symbols and convictions depend (Rappaport, 1999, p. 21). Social norms embedded in religious dogma dictate agreed-upon ethical forms of action, thereby helping manage the passionate behavior of individuals and groups. Norms can be passed down as part of the extended ecology and cast into practices, to such an extent that conformity to required public behavior is more important for social living than actual privately held beliefs (Hayden, 2003). Indeed, an unambiguous public act is fundamentally social and thus provides the basis for public order and obligations in a manner which the holding of private beliefs cannot. In addition to bold decree, conventions, rules, and norms are established through such performative acts (Rappaport, 1999, p. 123). The process of maintaining shared social norms as an extended ecological niche requires learning and evaluation, as well as confrontation and revision, but most importantly it is perpetuated through participation in ritual.

### Ritual and Logos

Ritual is “the performance of more or less invariant sequences of formal acts and utterances not entirely encoded by the performers” which “*logically entails the establishment of convention, the sealing of social contract, the construction of the integrated conventional orders we shall call Logoi (i.e., logos), the investment of whatever it encodes with morality, the construction of time and eternity; the representation of a paradigm of creation, the generation of the concept of the sacred and the sanctification of conventional order, the generation of theories of the occult, the evocation of numinous experience, the awareness of the divine, the grasp of the holy, and the construction of orders of meaning transcending the semantic”* (Rappaport, 1999, p. 9).

Ritual is the space within which the truth of our symbolic conceptions of reality are forged. These orders bind together natural, cultural, social, individual, group, discursive and non-discursive elements into a certified whole, a Logos (Rappaport, 1999, p. 346)^5^. We can observe the political nature of ritual and logoi for example in early manifestations of institutionalized ritual organization such as secret societies (Hayden, 2018).

As opposed to pathos (emotion) and ethos (honesty inspiring trust), logos is a mode of persuasion (Tallis, 2018). This sacred truth, or *mysterious intelligibility*, gives shape to our perception of the world, it provides an all-encompassing order not only of nature but also of morality and thus the ways in which we structure human society (Rappaport, 1999, p. 369). It does so through naturalizing self-consciousness, agency, selfhood, and intelligence into a mathematical order subordinated to the principle of sufficient reason regulated by laws and patterns of causation (Tallis, 2018). Logos is a source for explanation, it is the local well of established and learned metaphysical symbols, established through convention and performance. We pursue the need to develop a personal sense of meaning by engaging in epistemic foraging through the collective wells of meaningful symbols provided in our culture's myth, religion, and ideology (Tallis, 2018).

The experience of the numinous, i.e., immanence, is archaic. Religion may be most accurately conceptualized as the cultural technology whereby discursive conventions of the sacred canalize non-discursive mammalian affective processes in ritual actions within a liturgical drama (Rappaport, 1999, p. 390). Significances and emotional associations are strengthened through the collective effervescence of ritual participation which uniquely achieves the hermetic bonding of groups of individuals into communities (Durkheim, 1915). Ritual has positive effects on social bonding and affective state (Charles et al., 2021). The emotional state which emerges from group ritual is sometimes called *communitas*, it stands apart from rational forms of organization and profane activities of the society (Turner, 1969). Émile Durkheim claimed this experience is fundamentally social rather than individual, though its proximal result is the personally-felt sense of profundity which solidifies beliefs in the given liturgical tradition being enacted (Mazzarella, 2017).

Emotions become attached to shared systems of meaning, logoi, through conditioning, and tertiary-level cognitive processes such as symbols and language (Panksepp and Biven, 2012). The unification of the community through participation in ritual that enacts logos is a higher sense of meaning, of immanent certitude. Power is routinized through these displays. Rituals evolve to convey information about metaphysical beliefs through repetition and costly displays of commitment. Religion is a way to arrange the mind by organizing one's sense of meaning in life, meaning as relevance (or salience), and semantic meaning, toward the goal of self-regulating her emotions and cognition. Religion requires a theory of systems, cognition, and a relational sociology, one of the mechanisms to enact these beliefs is ritual (Oviedo, 2019). There are two types of ritual: imagistic ritual is a once-in-a-lifetime event, these initiatory ceremonies generally employ a dysphoric situation to bond the novitiate to the group and his new identity through trauma (Whitehouse et al., 2017). Doctrinal ritual, on the other hand refers to standardized, rote sets of behaviors, such as daily prayers (Whitehouse, 2015). Accordingly, rituals performed more frequently tend to become less emotionally stimulating (Whitehouse, 2000; Atran and Henrich, 2010). The reason particular ideas that provide a background for doctrinal ritual such as miracles and stories of the gods are memorable may be because they break expectations in such a way as to grab attention and remain memorable (Atran and Norenzayan, 2004). The costliness of embracing seemingly absurd beliefs is said to cement social bonds between believers (Atran, 2002). Ritual sanctifies belief, it shifts the world away from empirical, social and pragmatic concerns to contain powers beyond those of rationality (Alcorta and Sosis, 2005; Hammoudi, 2006).

The psychological state implicated in ritual participation engages a type of thought, *mythopoetic*, which is heavily imagistic and embedded in sensible affective modes of knowledge (Turner, 1969: 277; Asma, in press). Rituals are performances that engage mimetic practices of imitation, or overimitation, wherein participants may not understand the causal story of what their behaviors are leading to but nevertheless mimic the behavior of those higher in the social hierarchy, and thereby solidify social norms (Johnson and Earle, 2006; Bulbulia et al., 2013). One of the ways imitation is assured meaningfulness is through indices that are impossible to falsify and resistant to misinterpretation (Rappaport, 1999, p. 56). For example, individuals regularly engage in physical torture, scarification, tattooing and learning immense amounts of information as part of ritual participation. It is worth noting the importance of mimesis for the arts, as imitation of nature in the Aristotelian sense, as well as in terms of mimetic desire which leads to dramatic societal conflict (Girard, 1977; Drost, 1991).

Mimetic behaviors involved in ritual amplify components of formality, pattern, sequence, and repetition through the practicing of liturgical elements as well as more immediate synchronization of rhythmic drivers in autonomic function, as in music, chanting, and dance (Alcorta and Sosis, 2005). We know that synchronizing pulse rate, heart contractility, and skin conductance is positively associated with feelings of empathy, which are crucial for maintaining shared social norms (Levenson, 2003). Music and dance in these contexts can evoke similar emotions because the symbols included in the logos are learned through conditioning, they are communal elements of affective and conditioned meaning encapsulated in imaginative culture. For example, the Māori haka war dance consists of rhythmic group movements, singing, and mutual emotional contagion. In Arabic music, the sense of *saltanah* or *tarab* (musical ecstasy) occurs in a cohesive group setting in response to motivational sound symbols like *qaffla* (resolution) and *maqam* modulation (Racy, 2003). These examples which I expand upon in section Arts of Immanence, illustrate how physical ritual requires internalization of symbols, stories, and a metaphysical frame which can then be embodied in communal participatory practices.

Communal ritual invests objects, individuals, and ideas with emotional significance which in part creates or perpetuates a shared symbolic system that gives a valence to individual choices and motivates behavior. The conditioned association of such emotions as fear and awe with symbolic cognitive schemata achieved through these rites results in the sanctification of those symbols, whether places, artifacts, or beliefs. Because such symbols are deeply associated with emotions engendered through ritual, they take on motivational force (Alcorta and Sosis, 2005, p. 341; Dehaene and Changeux, 2000). The coordination of bodily affective arousal in individuals as well as across a group of participants engage non-linguistic drives which attain apperception of sacrality (Lex, 1979). “*Sanctity…stabilizes the conventions of particular societies by certifying directives, authorities who may issue directives, and all of the mythic discourse that connects the present to the beginning, establishing as correct particular meanings from among the great range of meanings available to the genetically unbounded human imagination*” (Rappaport, 1999, p. 321). Sacrality is akin to the feeling of immanence, it acts as a felt anchor to the legitimacy of belief in a logoi.

In this state of immanence, the postulates that tend to become ingrained in individual belief systems through this ritual process cannot be falsified or verified logically or empirically. They are felt to be beyond question and supported by a kind of sanctity that renders belief impervious to change through discourse (Rappaport, 1999, p. 281). There is a certainty beyond doubt that rituals engender through their engagement and synchronism of affective states; this is the context of belief fixation. Doubt, which arouses anxiety and seeks the closure of certainty is assuaged through rituals that establish logoi. This is the experience of the numinous: the non-discursive, non-rational, ineffable portion of the Holy^6^. In the modern era, we attain such certainty through the same means: ritual, emotional saturation, charismatic figures, and ceremonies of legitimacy. The intimate communication of reading, the absorption of viewing pictorial art at a museum, or the overwhelming experience of the concert hall and the nightclub all take place in spaces of enchantment. Art has taken on many of the practices and methods of ritual experience thus developing a creative use of psychological manipulation that allows for the contemplation of sacred truths of personhood and cosmology through beauty.

Central to the notion of imaginative culture is the relation between psychological motivation and social function. I argue that this relation is embodied in religious behaviors—most importantly, ritual—and art. Specifically, the proximate emotional causes and adjuncts of religion and art provide space for belief. Ritual creates, recognizes, and discovers, the sacred, specifically the sense of emotional significance which then motivates belief. “*Belief is…achieved through ritual behaviors…ritual presentations of myth, ascetic practices, and healing ceremonies, all of which instill an experience of…the ‘sacred'*” (Purzycki and Sosis, 2013). Spiritual emotions, like awe, create conditions for the imprinting of metaphysical beliefs. Participation in ritual disables emotions arising from fear and anxiety, thus mediating between conflicting drives as a form of sublimation (Rappaport, 1999, p. 49 n12)^7^. Whether through public displays of strength as in the military parade or public displays of wealth as in private museums or conspicuous consumption, the generation of awe functions as a tool for the legitimatization of power (Johnson and Earle, 2006).

The materializations of beliefs implicit in ritual can be shared by communities both synchronically and diachronically in works of art. Art materializes experience, bringing a cast of mind into the world in objects (Geertz, 1983). “*It is out of participation in the general system of symbolic forms we call culture that participation in the particular we call art, which is in fact but a sector of it, is possible*” (Geertz, 1983, p. 109). For example, so as to present the metaphysical beliefs of their community, shamans create stories, songs, and parietal art to materialize the mystic visions they experience through trance and altered states of consciousness (Lewis-Williams, 2002). Alterity and metamorphosis is central to the process of creating the sense of *communitas* in the overwhelming affective experience of immanence. Communal rituals which depict logos, or *mana*, then have a function of stabilizing social organization (Johnson and Earle, 2006; Mazzarella, 2017; Asma and Gabriel, 2019).

Imaginative culture plays a crucial role in recurrent aspects of religious behavior: belief in supernatural agents and counterintuitive concepts, communal participation in costly ritual, separation of the sacred and the profane, and the importance of adolescence as the life history phase most appropriate for the transmission of religious beliefs and values. Emotionally charged imaginatively-derived symbols invest objects and ideas with motivational meaning in the context of religion and ritual (Alcorta and Sosis, 2005). Art is a natural human activity (Dissanayake, 1992). Ancestor worship is implicit in ritual objects, for example wine vessels and bronze pots of the ancient Chinese Bronze age. These objects perpetuate the liturgical order of the time by embodying it in the object as awe toward mortality in nature worship or belief in spirits from the power of objects and spectacle. Additionally, well-crafted rhetoric convinces the listener about particular claims one may not have entertained; it is partly the art of the rhetoric that induces awe and other elevated emotions which push an individual toward revising her beliefs. Religious art clearly depicts mythic stories and allegories that underlie ethical precepts. The grandeur with which we see these parables depicted in the high period of Christian art in Western Europe in the last 2000 years, whether in the Cathedrals of Italy or the wooden votives of Prussia testify to the awe-inducing spiritualism of such objects. There is also the nameless sensation engendered by landscapes and abstract patterns and images which nudge the mind to consider mystery. Much of modern art seems to be engaged in materializing such a sense of confusion and alienation in the clattering plenitude of our times.

Saturated by the emotional experience and the consequences of alterity in the immanent encounter of imaginative culture, information made salient in the sacred experience can become the basis for beliefs (Durkheim, 1915). This is true both for the artist (or shaman) as well as the other participants who are compelled through the artistic object or event into spiritual emotive states wherein they become receptive to the fixation of metaphysical belief. Immanence implies unforgettableness, complete presence, this feeling serves as an anchor for the salience and perceived correctness of the belief associated with the event. Belief is not necessarily a reflection of truth, rather it serves as a reflection of the social sacred and its attendant forms of ministering to affective needs with the available tools/language games^8^. I provide examples and elucidation of these idea in section Arts of Immanence.

In the power of drama, we surrender and are changed^9^. The feeling of immanence experienced during rituals, carnivals, concert, dramas, and cinema act as liminal zones of time and space. Liminality and marginality are conditions in which myths, symbols, rituals, philosophical systems, and works of art are particular potent (Turner, 1969, p. 128). Good stories “in addition to the pleasure they bring us, serve to file down and better sharpen our judgment, such that pleasure does not remain pointless” (Amyot, 2008, p. 159). Thus art as a creative use of psychology sharpens our affective sense of reality through the symbols of the local knowledge practices. In these spaces, humans use mimetic representations to reflect on social structures, desire, and existential questions (Turner, 1969). Artists are responsible for constructing ways for the culture to affectively experience and consider the meaning of their local symbols. Art is thus a sensible affective knowledge practice that bestows self-knowledge through the play of symbols drawn from an epistemic niche.

## Creative Uses of Psychology in the Arts

### Art as Self-Knowledge

The arts consist of techniques to craft objects and ideas that play with emotions toward engendering transformations of understandings of space, place, identity, thoughts, and beliefs. Ranging from enjoyment to torture and reflection to laughter, pushing one to the edge of confusion or the fog of the sublime, the affective states achievable through art and ritual are accomplished through creative uses of psychology^10^. In encapsulating deeds of thought, art articulates humanity in a way that is informative as well as pleasurable, a *utile dolci*. As discussed above, this emotional element is crucial for how art practices engage belief. It is through artworks that we consider the human condition. Indeed, the imagination is a tool for wisdom, it aids in the development of macro-level, coherent, global explanations. Art allows for the creative discovery of connections by revealing multiple aspects of ideas and objects (Roszak and Berry, 2021). Thus, I discuss imaginative uses of psychology as forms of reflexivity.

Cultural conventions like ethical norms can be worked out through the mimetic function of representation in Art. This is because the way we depict ourselves, the way we talk about ourselves, our sense of norms all must be displayed in a form which complements its content. Whereas norms are often communicated didactically or prescriptively as dogma, they can also be adjudicated through simulations in fantasy worlds and in artful forms of expression. The aestheticizing of these fantasies can glorify and elaborate upon the ideas presented. The depiction of mythological stories, like those of Hercules, or the glorification of Oba in the Benin bronzes, serve to present norms of heroism and greatness. Our emotional lives are domesticated into symbiotic norm-governed relations in groups, through such social practices of imaginative culture. The social norms derived through imaginative culture are forms of affect management perpetuated through normative rituals and traditions^11^. The depiction of legendary figures, like Aeneas who founded Rome, allow for the concentration of affective power upon a symbol. It is through symbolic activities like these examples of public memory that people are adapted to live in culturally defined cooperative groups (Richerson and Boyd, 2001).

Art is a form of self-knowledge that equips us with a motivated understanding of ourselves in the world (Pippin, 2020)^12^. To achieve this requires aesthetic attending, an imaginative seeing of the work (Pippin, 2017). An artist will engage their audience's well of symbols, their cultural epistemic niche, through the capacity to gain acquaintance knowledge through aesthetic comprehension. Art is thus implicated in the “construction and deconstruction of conventionally constructed symbolic systems” that materialize experience and superstitions through formal content and can then be used by individuals to make sense of the things that happen to them (Geertz, 1983, p. 119). We observe this process for example in hyper-motion with memes that symbolically represent ideas and affiliations and can be (de)constructed according to various ideological platforms.

Along with philosophy and religion, Hegel considered art to be part of our collective attempt to gain self-knowledge. We learn what we really think about shame for example by attending to its display by Emil Jannings in *Der blaue engel* (Pippin, 2017, p. 4–6). The mimetic function of art in tragedy and cinema are then forms of moral psychology. As Aristotle would have it, we learn about ourselves, about our condition through watching others like us. For German Romantics like Friedrich Schiller, art is a means of self-completion through which we cultivate and ennoble human nature. The aesthetic condition achievable through art allows for the totality of human potential, partly through extending our notions of what is possible and learning from simulations of reality (Schiller et al., 1993). Similarly, for poet Rosanna Warren (2008), literature is that symbolic space in which we make formal, imaginative experiments in consciousness and conscience. Poetry is then “art in quest of difficult knowledge.” One technique by which literature accomplishes the experience of deeper meanings is the use of metaphor to enrich and offer affectively powerful resonances (Rappaport, 1999, p. 393)^13^. Analogical thinking is one of the main tools used by empirical psychologists to construct a narrative order out of disparate empirical facts (Gabriel, 2021b). The mimetic representation of mind through metaphor allows us to see ourselves under such description. This analogical aspect of scientific explanation functions along the same lines as mimesis in art: symbols structure the order we experience.

The function of art and ritual is not simply acceding to Dionysic affective states, it is in the service of the broader organization of discursive and non-discursive understanding. Art articulates humanity by rendering explanation through the techniques of immanence. Like ritual, or as ritual, art forges an image of the divine through language, movement, and the motivational force of the emotions (Rappaport, 1999, p. 388, 399). “If art and ritual, and art *in* ritual, are successful they construct “sentiments” out of the inchoate stuff of vital experience on the one hand and objects of discursive relation on the other.” They point toward grace, or holiness, that is, the reunion of the “multiple levels [of the mind] of which one extreme is called consciousness and the other the unconscious” by guiding experience to particular objects of thought (Rappaport, 1999, p. 387). In section Mimetic Arts, I explore the power of the arts to compel belief fixation through emotional states of immanence using examples from tragic drama, cinema, the sublime in visual art, and the use of music to engender trance states.

The communication at the core of artistic practices is special not because the artist has something to express, but because what she has to express can be shared. We are sense-making animals and thus we must be attentive to the ways in which artists lead viewers to make connections. This manner of manipulating our proclivities to form particular understandings is a fundamental aspect of the shared experience of art. In viewing a work, we acknowledge the artist's view of the world, we thus enter into an alterity: identification with another imagination within the context of our own shared and unique point of view. Such a conversation quite often leads to new knowledge about the self, society, customs, ethics, techniques, and aesthetic objects. The arts are capable of establishing mental relations and associations between and across different natures through a symbiotic alliance; as a heterogeneous alloy of methods and mediums, we can call them assemblages (Delanda, 2016). Thus, the creativity assemblage is a ‘line of flight' that allows affect to flow between ideas, bodies, and things^14^. What is the nature of the knowledge derived from and through the arts?

### Sensible Affective Forms of Knowledge

The format of mimetic and immanent arts is not the same as discursive or descriptive analysis, their methods are culturally evolved for the purposes of exploring and deriving other kinds of knowledge. I argue these elusive forms of knowledge are intrinsically tied to emotions, the body, and the imagination. Philosophy is a discursive rational project, while art seeks knowledge through engaging the affective, sensory modality. For Hegel and the German romantics, like Schopenhauer on music and Nietzsche on tragedy and Wagner, art can be a form of collective self-knowledge in which humans externalize and self-realize their conceptions (Pippin, 2020). A Hegelian approach treats artworks as “rendering matters of concern more intelligible to us in a distinctly sensible affective way—treats artworks as instances of determinate, and certainly not accumulating, knowledge claims or as evidence or justification for knowledge claims” (Pippin, 2014).

Hegel deepened Kantian notions of the kinds of content that can be communicated through art^15^. He claimed that sensible affective self-understanding is itself a form of insight that is philosophically and historically sensitive. For Enlightenment thinkers, art was a way of relating to the world, a free play of faculties and non-cognitive recognition of the purposiveness of nature relative to the moral nature of humans as free beings. This type of knowledge was said to not be eclipsed or replaced by rational processes, yet we have not been able to clarify what such knowledge consists in.

One approach to specifying this knowledge is to focus on the aesthetic dimension, namely how beauty allows us to understand the reality of freedom in the world (Pippin, 2014, p. 7–8, 11, 13)^16^. Another tack is to interrogate the art object as to the practices behind its production. This leads to appreciation of its existence within the social order as part of an historical materialism (Clark, 1999). The latter approach enables us to understand ourselves in the context of our historical moment through the art object. This amounts to using idiographic factors to determine how the intention of the artist is realized in the work as a representation of the sensible affective profile of the community of which it is a product. Rather than pursue psychic laws of causality, the humanities are concerned with questions of interpretation, aesthetic judgment, and ethical evaluation (Rodowick, 2015).

These aesthetic and materialist approaches are couched in Hegel's larger project to identify how *Geist*, the collective spirit, comprehends an age in thought by assessing the rationality of its norms (Pippin, 2014, p. 20, 24). In this scheme, art is the way humans realize freedom by establishing a self-understanding that aligns rational social relations within the context of the community. For Charles Taylor (1971), interpretation and understanding require a reflexive turn in self-interpretation because knowing is intertwined with questions of value and significance. Hegel claims that *aesthetic intelligibility* is a modality of sensible affective truth, or genuineness. Artful expression is then a form of philosophy (Rodowick, 2015, p. xv.). This all depends upon our intelligibility to each other as social agents within a collective cultural niche of symbols as well as the maintenance of the following qualities in an artwork: credibility, compellingness, and conviction (Pippin, 2014, p. 135).

The broader Hegelian project is not essential to my argument, since aspects of other approaches such as that of Aristotle similarly cohere with my project. For my intents and purposes, what Hegel means by *Geist*, I take to mean the power of reflexivity: how we can represent ourselves to ourselves within a situated system of symbols. The study of mind which has come to be called psychology in the last 150 years is in essence a reflexive project wherein, using the symbols of a secular, technological society, we question how and why we think the way we do. I would like to suggest that preceding and coinciding with the project of empirical psychology, artists are also engaged in a study of the mind^17^. Yet their goal has never been nomothetic, that is, to create general laws, rather artworks are created as idiographic reflections, crystallizations, interpretations, judgments and ethical considerations about particular local cultures, and, sometimes, something like truths about the human condition.

My unique contribution to the question of how art enables self-knowledge is an elaboration of those aspects of mind in between intuition and conceptual thought that actually mediate sensible affective knowledge. I emphasize the role of sensation-perception and the imagination in these intermediate forms of knowledge^18^. I also explore how knowledge by acquaintance is another sensible affective mode crucial for the arts of immanence in which first person acquaintance provides authority for fixing belief (Lewis, 1989). Crucially, sensible affective knowledge is largely contingent and context dependent, and yet this reliance upon context and contingency is precisely what allows it to provide knowledge that nomothetic projects cannot (Rodowick, 2015, p. xi, 301)^19^.

It is possible to explore the mechanics of sensible affective knowledge now because the study of emotions in recent decades has transformed our understanding of their range and capacities (See for example, Panksepp, 1998; de Waal, 2001; Prinz, 2004; Phelps, 2006; Davidson et al., 2009; Pessoa, 2013; Damasio, 2019). Meanwhile, ecological psychology (also referred to as direct perception) has refined our understanding of the active nature of sensation and perception (See for example, Turvey, 1992; Reed, 1996; Chemero, 2009; Withagen and Chemero, 2009; Heft, 2010; Rietveld, 2012). Affective neuroscience and ecological psychology both arise from reflection upon the insights, and shortcomings, of prevalent methodologies in the cognitive sciences, social psychology, and machine learning. This allowed for shifts in focus, from cognitive to affective aspects of mind and from computational mechanics to bodily–perceptual explanations of behavior. Emotions play a role in aspects of mind heretofore misunderstood as simple and passive. In fact, emotions, perception, and non-linguistic imaginative processes are together capable of substantial adaptive feats. Elaborating on sensible affective knowledge can be done by drawing on elements of direct perception, affective neuroscience, and the evolution of the imagination.

### Emotions and Direct Perception

Emotions are most accurately conceived as affective systems hierarchically structured in layers of interpenetrating functions and motivations (Panksepp, 1998). The evolution of mind is the developmental story of how these layers emerged and acted as feedback loops on each other. Such feedback is an embodied, enactive, embedded, and socio-cultural process. Imaginative culture is conditioned into our thoughts and motivations through the cultural lifeways that make up our epistemic niche. The indelible richness of experience is formed of such associations that weave together multiple strands of memories, emotions, ideas, and glimpses which range from bold presence to ghostly whisper. It is only with great difficulty that we attempt to trace the complexity of our conscious experiences, and that is why we rely on knowledge from acquaintance upon which we can say we know something because we are conscious of it. In this context, language is given epistemic legitimacy because it allows us to say something even when our statements do not convey fully (Wittgenstein and Anscombe, 1997).

In the evolutionarily oldest and anatomically lowest part of the brain, are instinctual drives, like fight or flight. Primary-process emotions include (i) sensory affects (sensorially triggered pleasant-unpleasant feelings), (ii) homeostatic affects (hunger, thirst, etc. tracked *via* brain-body interoceptors), and (iii) emotional affects (emotion action tendencies). The primary layer influences the secondary-process emotion, which includes social emotions, like GRIEF, PLAY, and CARE. This layer is sculpted by learning such as classical and operant conditioning as well as habits at the secondary level and then by language, cognition, and regulation at the tertiary level. Emotions are thus capable of learning, of complex admixture with memory, and various forms of domestication into social and artistic functions^20^. There is thus a capacity for knowledge in the emotional system. Yet we do not have clear introspective conscious access to the functioning of primary and secondary layers, they are often unconscious. Our kind of consciousness is primarily mediated through language and cognition, as well as the tertiary-process emotions into which we are acculturated^21^. Unfortunately, this was the only layer that most philosophers and psychologists took into consideration due to Cartesian and anthropocentric biases. That is why it has been difficult to conceive of sensory affective knowledge, because knowledge was taken to only mean propositions or other declarative information.

We know now though that there are plenty of things that the body knows that cannot be stated propositionally, i.e., procedural or muscle memory, such as how to ride a bike or how to dance the mashed potato. Direct perception is a school of empirical psychology which describes how perception–action systems, like visual or auditory senses are constituted of relational loops with the environment wherein sensation produces dispositions to act in the perceiver (Reed, 1986). Effectivities provide the animal's bodily dispositions in relation to the perceived surface, or that which seizes and actualizes affordances, which are the indicative and imperative elements of the sensory percept (Shaw, 2003; Withagen and Michaels, 2005). The imperative aspect of a percept dictates how the animal can respond, for example spatial navigation relies on local cues that designate possible routes (Millikan, 1996). An actor chooses affordances to be realized, a corresponding mode of action, and the appropriate laws of control by which to regulate the action (Warren, 1988). An organism's exploration of the environment includes prospective control, that is, modifying its relation to the environment to perceive particular affordances in the context of goals to be realized (Pezzulo and Cisek, 2016).

In addition to physical ecology, we can extend the notion of the niche occupied by humans to its sociocultural constituents, the epistemic niche of symbols and other social actors. From neonatal care relationships to coalition-building, to apprentice learning of sophisticated skills to cultural institutions, other social agents are an intrinsic part of the environment. Interaction takes place in a niche wherein communication and social position provide affordances for social navigation (Warren, 2006; Laland et al., 2010).

The elements of direct perception which may mediate sensible truths when applied to the aesthetic intelligibility of art are: affordances, effectivities, imperative representations, social and spatial navigation, and emotional contagion. These processes are all hooked in to affective reactions to the environment in the context of an organism's pursuit of endogenous homeostasis (Damasio, 2019; Gabriel, 2021b). Emotions are manifested as evaluative and expressive actions or dispositions toward objects in the environment. Emotional contagion, the sharing of emotions *via* non-verbal means, is widespread in mammals and begins in infancy in humans, for example could be the basis of empathy unto feelings of alterity and metamorphosis that occurs in the mimetic arts and the shared ritual experience of performance (de Waal, 2001).

The crucial connection between sensible and affective knowledge is the imagination: a precognitive simulation system that mediates between perception and cognition. According to Thomas Aquinas, imagination is an expanded perception of the world, it is necessary for abstraction of material from the senses and thus determines the quality of cognitive processes (Roszak and Berry, 2021). For instance, imagination is the sphere of playful adumbrations of image, sound, and movement. Voluntary imagination evolved from earlier involuntary imaginative states such as dreams (Panksepp, 1998; Asma, 2017). There are other evolutionarily early forms of imagination which produce knowledge that is not declarative but rather poised between concept and intuition. They include: image-based inferential processes, image grammar, body-task grammar, non-conceptual content, unconscious bundling, prototypes, and analogical modeling^22^.

Various forms of art are thus creative uses of psychology that attain knowledge through sensible affective means. Sensible affective means consist of the tripartite affective system, direct perception, imagination, and knowledge by acquaintance of immanence. To demonstrate these connections, I group the arts in two formats: (a) Mimetic arts (such as drama, cinema, and literature), in which individuals empathize into an alterity and may undergo ethical transformations through metamorphosis in the process, and (b) Arts of immanence (such as music, dance, painting, and cinema), in which emotions learn through the entrancing imaginative play of immanence.

## Mimetic Arts

### Drama and Literature

Plato tells us in the *Republic* that art is imitation, that the artist holds a mirror up to nature (Harrison, 1913). We know it is more complicated than that, that mimesis is a moral form of imitation, that the manner in which an artist renders nature is through his aesthetic education and the intentions brought to bear on the task. We also know that imitation is a form of bodily identification in which we internalize and mirror what we observe. Let us review some forms of imitation involved in the practices of art. Art and ritual share the mimetic impulse to express “a strongly felt emotion or desire by representing, by making or doing or enriching the object or act desired” (Harrison, 1913, p. 27). Viewers are drawn in through emotional contagion and the activity of mirror neurons (Gallese et al., 1996). Seeing another person cry or erupt in a rage is an imperative representation, it forces us to go along with the emotion being expressed or actively ignore the impulse (Millikan, 1996). Such affordances and effectivities abound in mimetic arts. Actors in theater in particular are trained in simulating recognizable social behaviors and forms of bodily and vocative expression to draw the viewer into the situations they are representing. These sensory perceptions of actors and characters can be ratcheted up to levels of great complexity when symbols and words are also loaded with emotions and associated ideas through the developmental process of acculturation. That is one of the reasons that art is such a potent tool for political expression and ideology (Trotsky, 1960).

The traditional manner of presenting mythology through oral recitation was expanded into dramatic tragedy and, subsequently, the novel. Each development allowed for further complexity and reflexivity between the work, the viewer, and the artist. Whereas ritual specifies a liturgical order, Greek tragedy elicits reflection upon that order by the audience (Rappaport, 1999, p. 42). According to Jane Harrison, drama comes from the Greek word for rite, “dromenon,” meaning “thing done,” and denoting religious ritual. Dramas like the Greek tragedies were an enactment of myth, which, unlike ritual, did not indicate its acceptance *per se*^23^. Ancient Greeks fashioned Tragedy to incite mimesis in an emotive ritual about social norms and the nature of compassion. Through engaging the emotional states of guilt and shame, characters in tragedy provide a focus for social expectations to consider how actions alter relations to the world. Actors do not take actions, rather they imitate action, which is then observed by the audience who enact a mimetic contemplation, as ritual participation in the drama (Rappaport, 1999, p. 137). In Greek tragedy, the viewer must locate a source of necessity beyond divine order and physical constraint in the internalized other (Williams, 1993)^24^. The genuine social reality of the limitations of metaphysical freedom are thus dramatized in Tragedy. This is especially clear in Attic tragedy which addressed crises in the basic institutions of society, for example, justice in Aeschylus' *Oresteia*, or the conflict between filial piety and civic responsibility in Sophocles' *Antigone*^25^.

The novel is comprised of subgenres which create models for mimetic reflection: a celebratory, idealist view of human life in the epic and chivalric stories, and a derogatory, anti-idealist attitude in picaresque stories, and novellas (Pavel, 2013). The nineteenth century European Novel refines drama into more involved narrative elements (Bruner, 1991). During this period of positivist optimism, and urbanization/industrialization, Honoré de Balzac and Emile Zola sought to provide a mythic description of their times. They achieve this through reflections on society and the role of the individual within it (*La Comédie Humaine* and the saga of the *Rougon-Macquart*) (Pavel, 2013). Rather than reduce lived reality to laws and processes as the empiricists were wont to do in their nomothetic practice, the project of the novel is stoutly idiopathic with the belief that such collective portrayals of individuals deliver the human form. Their exhaustive descriptions maintain an air of the descriptive project of humanism. As if to say that if all material and mental conditions were laid out empirically and phenomenologically, then a complete description could constitute something like an explanation. Expressivist poetry and diarists like Jules Renard in his *Journals* likewise transcribe the man to himself. The great novelists of the nineteenth century were chroniclers of the human heart. Indeed, the novel provides a format for writers to assay the major conflicts of their time in the form of increasingly self-conscious narrative structures in which literary technique depended on changes in the social structure (Watt, 1957). Literature allows for narratives that explore how individuals relate to the society in which they live, with all its tensions, aspirations, and constraints (Lukacs, 1916/1971). Readers mimetically sense the conditions through the rhetoric of realism and the artful presentation of the situation rendered by the writer and elaborated upon by their own imagination and sympathy. The ambition and popularity of nineteenth century literature suggest that it provided a superior way for the artist and the reader to consider the world they lived in. Ethical transformations could take place through this encounter, for example Upton Sinclair's depiction of the slaughterhouses of Chicago in The Jungle (1906) raised for public reflection the conditions of industrial animal sacrifice.

Knowledge by acquaintance is a rich tool in the artist's arsenal. It can be achieved for example in how memory is regained through the artful exploration of thought, nostalgia, and ethics. From Marcel Proust to John Milton, a paradise is rendered in the *utile dolci* of imagined worlds both informative and pleasurable. Moral literature aims for such a convergence. There is a mimetic function played by literature in this sense, it provides a simulation of the social imaginary in which we can conceive of our ethical pursuits (Dutton, 2009). Because narratives enact ideals and norms, they propose hypotheses about human life and imagine fictional worlds in which characters like the readers exist with qualified autonomy. These imaginative projections include the depiction of human types and consideration of the meaning of life, love, and the nature of human interaction (Pavel, 2013, p. 17–19). These elements of the modern novel dovetail with the reason we derive intuitive satisfaction from modern psychology, namely because it furthers core values of expressive individualism and voluntarist enlightenment humanism. From the epic to chivalric tales, tragedy, and a vast array of narrative formats, imagination is our omni-host in the mimetic arts. In this way, mimetic arts engender a sense of immanence through garnering personal and epistemological significance as narrative forms in the context of secular modernity. We understand ourselves and others through the self-reflective experiences of attending to the dramas that invite mimetic participation.

### Cinema and Ritual

Myth and ritual are central to how every society collates, creates, and perpetuates the core of its significances, values, aspirations, origins, goals, and ethical lineaments. Rituals occur in the enclosed space of the ritual arena. In these spaces, different rules apply. They bring about different ways of relating to the community, to ancestors, time, and fundamental notions of meaning and significance (Heesterman, 1993). Rituals are a form of communication beyond language, they carry canonical messages which are difficult to fake or misinterpret for members of the community (Rappaport, 1999, p. 140). Ritual makes facts palpable through their performance. It is thus illuminating to consider how the arts constitute a transformation of these functions of ritual and myth (Nugent, 2017). The two communal rituals I focus on are cinema and deep listening to music. These formats of mimetic and immanent arts allow for the derivation of sensible affective knowledge.

Cinema has become an effective medium for portraying complex stories that enable viewers to participate in, and reflect upon, the myths of our times^26^. In movies, the scale of human action takes on mythic proportions. Not only in size and effort and money necessary for production but in terms of the importance of gesture, storyline, character, mood, setting, and scene construction. Archetypal characters, or types, bring to life finely-wrought scripts wherein storylines illustrate moral reflections, aspirational narratives, and the dramatization of historical events. Patterns of social action are represented in the form of a story grounded in aesthetic and sensory perceptual forms, historical settings, and interpretational structures (Rodowick, 2015, p. 25). By presenting ideas in this manner, film provides an epistemological platform for our ethical consideration. The ways in which this is materialized can take an almost infinite range of forms. To take one example, Alfred Hitchcock's Vertigo (1958) brings the audience to consider our mutual interpretability: how to make sense of each other's motivations in the broader context of our lives and desires (Pippin, 2017). John Ford's The Man who shot Liberty Valance (1962) provides a way to understand how the Law is established, how a city comes to be founded through the ambiguity of moral claims. The character John Wayne plays in the classic Ford Western is an epistemological foundation to consider the uses of violence, coercion, liminality, foundation narratives, the wild West, and many other issues in political theory (Pippin, 2012). Cinema is not repetitive nature, rather it shares with ritual the endlessly iterative production cycle of the bardic griot recitation of mythology^27^.

Cinema regularly induces a sense of immanence in the viewer through several means; it takes place in a mythic time of heroes (Bogart, Garbo, Brando, Grodin) and is enacted in an immersive atmosphere not unlike the ritual arena^28^. The movie theater, a closed room, dark but for the light burning through translucent celluloid, in which we sit among strangers with whom we share emotions to spectral events in “speculative solitude” (Cavell, 1979, p. 7). By sharing our reactions, we consider and often reinforce cultural values. The communal setting of the ritual arena of theater confirms us as a community and builds our shared history and interpretation of reality out further. Light flickers on the empty frame, which screens the viewer from the world and the artifice of the film from the viewer (Cavell, 1979, p. 24)^29^. Cinema is larger than life, that is how it can represent life to us in a mimetic ritual of drama. We understand something about ourselves by becoming other or anonymous, by being taken in by the story and coming out the other end in a slightly altered form.

Then there is the narrative, the aesthetic charm poured into the dramatic form. That distillation of life into moments, sieved into distinct memorable tales. This narration by the camera, the filmmakers, the unity of interpretive meaning behind the selection of shots, give cinema its reflective form (Pippin, 2020, p. 24). The overwhelmingly melodramatic format of the journey, the love triangle, romantic love and skepticism, the conflict, the play of trust, etc., pushes us to identify with the characters. Some movies bring us into the community of the characters such that plot development resembles nothing so much as gossip. This mimetic form of interaction with fictional characters allows the viewer to enter into the social situation, to think of her reactions, her proclivities relative to the actors on screen. For example, In Jean Renoir's La Règle du Jeu (1939), one is led to consider the decadence of leisure, the fickleness of fun. We are brought into empathy and ethical reevaluation by considering human motivation in diverse moral contexts (Pippin, 2020, p. 14). Knowledge is here mediated by our emotional reactions, while the ways in which we derive meaning from the perception of moving pictures rests upon our shared epistemic cultural niche of symbols. The characters, the sets, the situations; if we are appropriately enculturated, they will be motivationally loaded with meaning by conditioned associations. Each symbol sings a note distinct. They draw out learned emotional reactions and lead us to make inferences. Emotions force us to question, to reconsider, to decide. They provide a non-conceptual content that motivates thought and action.

We accept film reality so easily because we already take reality to be drama; in fact, it may have to be dramatic to be convincing to secular moderns (Cavell, 1979, p. 90, 92). The actors, sets, and script by being recorded and presented in a ritual arena convert profane time into mythic time. The process resembles a rite of sacrifice—profane time becomes immanent through the medium. Dialogue in movies is so finely selected that it resembles the manifest content of dialogue in dreams, which Freud claimed was of utmost importance since the medium of dreams is visual such that words that sneak through the censor must be vital. Cinema presents dream and desire in the most accessible of formats, for it is in dreams that we lie in the dark watching moving pictures over which we have no control. But cinema, like dreaming, is more than an illusion, it can be a mode of reflective thought through image-based inferential processes.^30^ Montage itself relies upon our ability to make quick unconscious inferences that give disparate images a narrative structure. For Deleuze, the movement-image, which is a long shot, is intercut with the action-image (the medium shot) and the affect-image (the close-up) to produce affection: “motor tendency on a sensible nerve” (Deleuze, 1986).

For example, we develop image-based grammar, like prototypes of characters based on physiognomy. Slight cues, like the shot of a building or the shadow over a face compel us to refer to mnemonic schemas and scripts, like the femme fatale or the lonely drifter, the one-horse town or the bustling newsroom. Unconsciously, we bundle images together as situations, settings, forms of life, and draw inferences thereby. The mind is thus engaged in analogical modeling, wherein connections are made between images and sounds across memories of other films and lived experience. The emotional associations that arise then cue us to the mimetic relation between cinema and life, forcing both conscious and unconscious reflections. From romance to violence, through cinema we learn prototypes of possible encounters that sculpt how we face our futures.

Time is transformed in the cinematic state; for us, it is an escape from private fantasies and responsibility, a vacation, a portal out of the world and its uncontrollable physical laws. The liminality of being out of time in a dark room seemingly “doing nothing” allows for separation from so much that binds us. We are freed from speaking, from the dense maze of active interactions that make up public life. Here we are voyeurs who participate emotionally, even when nothing is asked of us. The situation depicted in the film becomes present for us, immanent in its richness because the filmmakers have fashioned it so. Through montage a story comes together, through casting we are directed to recognize characters, through lighting and sound we are immersed in environments. We spot prototypes of narrative arcs that draw our expectations and thus our further participation in the immanent art. All we have to do is keep our eyes and ears open. If we give it attention, the screen creates the illusion of four dimensions. It offers them to us, it takes them all up and drags us in its wake. The emotional intensity of the ritual, the sense of presence, of the immanent encounter with the mythic, draws from us the ingredients necessary for belief fixation: salience and profundity, credulity. “*It is the knowledge…that we exist in the condition of myth: we do not require the gods to show that our lives illustrate a story which escapes us; and it requires no major recognition or reversal to bring its meaning home. Any life may illustrate any; any change may bring it home*” (Cavell, 1979, p. 157).

After the liminal, anonymous space of the ritual, we leave the arena, discombobulated, and sensitive to light and motion. We reaggregate ourselves after having imaginatively become part of a different reality. After the affectively disruptive ‘line of flight,' we reflect upon what we saw and heard. The movie becomes memory, remembered as if in a dream (Cavell, 1979, p. 12). We return to each other loaded with stories, characters, and ghostly memories. The myth has been received, the experience has become a part of us. The affection-image is both reflecting and reflected; it is a combination of immobile unity and intensive expressive movement that refracts through us as affect (Deleuze, 1986, p. 87). Cinema offers moments of alterity in which we are transformed through the power of imagination.

### Arts of Immanence

Art is linked to ritual by their shared methods for heightening the intensity, or sense of immanence, of a situation. Participation in rituals and aesthetic attendance to various forms of art engender: (a) states of alterity in which we lose ourselves, and (b) emotional experiences that transform our belief states. In addition to image-based inferential processes, sensible affective knowledge engaged by art often relies on the feeling of immanence as discussed in section Cultural Evolution. This knowledge by acquaintance can lead to changes in an individual's beliefs because the emotional drama of entering mythic time within the shared space of the spectacle is one of the most powerful experiences an individual can have^31^. I will not endeavor to specify the exact knowledge attained through these practices, but rather suggest how art enlists the processes that constitute sensible affective knowledge to bring them about.

The role of participatory ritual, i.e., Dionysian ritual, is to edge us toward a state of receptivity, of liminality, confusion, emotional saturation, and maybe most importantly, a state of surrender. In surrendering ourselves to become completely present, we often refrain from rational contemplation, language, and prospective memory. In the arts, one may focus on the spectacle, the dance, the sounds, the cinematic screen, to the exclusion of all other thoughts and external stimuli or bodily action. This attentive state sometimes allows for absorption unto a sense of immanence in the viewer; a state in which stories, characters, environments, emotions, and ideas become powerfully present.

There are many methods by which such absorption can be elicited—from lighting to assaultive sounds to transfixing actors and actresses, compelling narratives, etc. Participation through attention and belief are the key to engagement in ritual. The quasi-sacred spaces in which art articulates cultural dramas of meaning resembles the arena of sacrifice. The concert hall, the movie theater, the church, the after-hours club, etc., all direct our attention and through their extended ecology provide a context for salient emotional responses. This bodily tacit knowledge frames our interactions and primes us for particular emotional experiences and social scripts.

With effective execution, art allows participants to lose themselves and come to exist inside, or alongside the spectacle. Drawn in its train, one becomes entrained to the unfolding display. The sense of time for example seems diffuse when one is in the flow of an engaging spectacle. A great movie or song or painting takes us in till we merge in communion. The ultimate forms of immanence are when an individual experiences trance and possession. Many musical traditions are built around such experiences; for example Arabic *tarab*, Moroccan *gnawa*, Tunisian *stambeli*, and American *rock n' roll*. *Tarab* is a borderline state of consciousness in which sensory and aesthetic percepts are mixed to afford a form of ecstasy, it is analogous to Spanish *duende*, Sufi *hāl*, and secular Deep Listening (Racy, 2003). These experiences resemble the possession states directed by shamans and rituals of spirit possession (Lewis, 1989). As imaginative uses of culture, they fulfill a similar function of solidifying metaphysical belief through spiritual emotions.

Analogously, in pictorial art a painting can arrest time and make present, or immanent, aspects of human action and of our condition relative to objects and nature. The way we see and the moments in which our nature reveals itself is the territory of pictorial art. In the aesthetic experience, one enters a state of reflective play in which pleasant sensations accompany aesthetic attendance and reflection upon the work (Pippin, 2014, p. 4 n8)^32^. Photographs, with their connection to the journalistic, truth-telling project, likewise capture light and time to bring the moment to us in all its immanent glory (Pippin, 2014)^33^.

Artists habitually report the sensation of being absorbed in the flow of the performance or creation of the art object. They experience a state of immanence in which their sensitivities are transformed; this is manifested in changes to their relation to time, space, other people, and the rehearsed or improvised content of the performance (Tan, 2018). Often, a sense of profundity ensues; it is fecund and leaves traces worth consulting for the creation of further work or in re-assessing the nature of performance. “(*M)ystical states seem to those who experience them to be also states of knowledge. They are states of insight into depths of truth unplumbed by the discursive intellect. They are illuminations, revelations, full of significance and importance, all inarticulate though they remain; and as a rule, they carry with them a curious sense of authority for after-time*” (James, 1910/1982, p. 380–381).

Participation is a sort of trade: time, credulity, and attention are sacrificed on the altar of immanence. It is a state in which one is rendered amenable to transformations in the form of emotional crises that lead to reconsideration of beliefs. You sacrifice yourself, your memories, preoccupations, and sense of individuality in the ritual toward a state of immanence in which truth seems to shine through. Escape is a type of sacrifice because you stop inhabiting your particular world, with its frames, habits, and familiarity. You escape *into* another world, you enter the mind of the people who produced the spectacle and, while it lasts, this imaginary world is overwhelmingly present. One can become other by identifying with characters on the stage, one could become other by losing herself in the world that the spectacle creates. This metamorphic merging with the spectacle constitutes a type of alterity in which forms of mimetic empathy, built upon emotional contagion, transform our notions of others and ourselves relative to them.

### Music and Trance

Sensible affective knowledge is achieved in the immanent arts through the play of imaginative faculties upon the individual's *habitus*, i.e., epistemic cultural niche. Trance is an endpoint of immanence, it is a dissociation characterized by a lack of voluntary movement and by automatisms of act and thought exemplified by hypnotic and mediumistic conditions. Adepts claim that riddles are solved and crises are resolved through the catharsis of the phenomenological space of trance. Individuals experiencing trance surrender themselves and thus enter a state of involuntary imagination in which, mediated by perception and cognition, their *habitus* is used as the material for the play of images, memories, neural-somatic states and image-based inferences. This is similar to the dream state in which symbols loaded with affective content populate involuntary image-based thinking.

Some likely sensible affective truths that emerge include shifting the emotional content between symbols, or exaggerating/diminishing the significance of a symbol depending upon particular contingent experiences the individual encounters. Part of the knowledge that is produced is a self-consciousness of one's context. This reflexive illumination arises from the heightened emotional saturation of the experience of entering a mythic time beyond the prosaic. There is also the mimetic empathy of communal participation in which our emotions are tethered to other people. The affordances made available in social spaces like dance festivals thrust one into a state of immanent acknowledgment of oneself as a body in space related to other bodies. Our spatial abilities are piqued by this non-verbal experience in which body task grammars are engaged (Barton, 2012). Social navigation is also activated in these situations and can also eventuate in changes in the evaluative tenor of social memory (Fiebich, 2014).

Trance is an altered state of consciousness that plays an important part in the ecstatic religious tradition. It asserts the confident and egalitarian relation between man and the divine (Lewis, 1989, p. 179, 184). Techniques for achieving trance include: alcohol, hypnotic suggestion, rapid over-breathing, inhalation of smoke and vapors, music, dancing, and imbibing psychoactive drugs. Sensory deprivation and hypnosis have also been implicated as inducers of trance^34^. Shamans and holy men regularly display their mastery of spirits by introducing them into their own bodies during trance states^35^. The mystical aspects include feelings of profundity which lead to the adoption or strengthening of belief, trance is accordingly often described as divine possession (Bourguignon, 1973). Examples of such practices can be observed in Nubian *zar* ceremonies, Haitian *voudoun* communities, Sufi *dhikr* ceremonies, as well as Pentecostal traditions of speaking in tongues. The sensible affective knowledge derived from such liminal states is directly dependent upon the public and socially-sanctioned shared symbols of the trancer's culture (Jankowsky, 2007). In this way, trancers enact the dominant beliefs of the community through being possessed by them in the ritual arena. These events serve therapeutic purposes as well; the possessing personality forces an identification with the trancer in the light of his personality needs, life situation, and cultural background to either act out a wish fulfillment or directly manipulate other people.

Music is an art of immanence which exteriorizes and socializes trance (Herbert, 2011)^36^. It has the capacity to engender trance through several characteristics, including rhythmic entrainment of autonomic nervous system (ANS) arousal and subsequent structural coupling between the individual and other social agents in supra-individual cultural syntheses (Becker, 2004). Certain emotional states like “chills” are mediated by endogenous opioids and oxytocin which play a role in social bonding (Panksepp, 1995). These characteristics provide excellent grounds for sensible affective knowledge in the form of direct perception and imagination. Direct perception of sound makes a body move; whether it is dancing, swaying, or launching into participatory sound-making, the affordances are present in the sensation of hearing. The absorptive state of sound-induced trance allows for imaginative involvement and hypnotic suggestibility that helps one transition out of prosaic time and into mythic time. This includes an escape from the bounded Cartesian self that makes possible a radical reflexivity (Becker, 2004, p. 27; Herbert, 2011). Other neurotransmitter systems, like monoamines and peptides implicated in such arousal states, seem to have an effect on one's sense of self, expanding it out and into the world. The skill of listening allows for all this to lead to emotional arousal, depersonalization, amnesia, and cessation of inner monolog (Becker, 2004). The autobiographical self may be occluded in trance by spirit possession or reversion to a more basic core bodily self (Damasio, 2012). What might be occurring is that bottom-up arousal is being interpreted by top-down cognition which contains learned social and cultural symbols. Trance and other forms of immanent participation are the space for the play of cultural symbols in which image-based thinking predominates. The transformations in notions of significance within the individual's system of symbols can be modified by unconscious bundling when the autobiographical self is re-appointed after the liminal state of trance. Fundamental beliefs are often drawn up in and around these cathartic reflexive experiences, which are altered states of consciousness.

Indeed, trance requires both psychophysiological modification as well as cultural materials. It has been used for a wide range of purposes, including modification of metaphysical belief (Rouget, 1985). We are physically susceptible to these receptive states and subsequently build cultural institutions like venues, festivals, and schools, around the immanent arts to produce artists who can attain trance states as well as generate them in others. These individuals, such as the Egyptian Sheikh Yassine il Touhami, generally straddle the social distinction between artists and spiritual teachers. Like shamans before them, masters of trance fulfill a crucial function for metaphysical belief fixation.

Beliefs are constructed out of cultural material and learned dispositions inhabited by the individual in the community (Bourdieu, 1984). For example, in the late eighteenth Century, through music, light, hypnotic suggestion, and creating rituals that leaned on his status as a magical and learned man, Franz Mesmer was able to induce trances in salons across Western Europe. French psychologist Jean Charcot and Pierre Janet likewise used their knowledge, educational status and other tools of authority to engender trance states in their patients toward exploring liminal forms of knowledge and memory of trauma (Becker, 2004, p. 14–16). Of course, in our times, it is psychologists and magicians who have a license to hypnotize us into trances and modify our beliefs about our cognitive and behavioral palette.

## Conclusion

In the state of immanence produced through the arts and in religious acts, the sense of meaning becomes profound, i.e., noetically distinct, because affect infuses the experience, and one's memory of it, with salience (James, 1902; Gabriel, 2021a). The quality imbued thereby makes humans attentive to subtle signs and broad “truths” (Bellah, 2011). We have a cognitive instinct to know and an inner instinct for self-preservation which is expressed as feelings (Schiller et al., 1993). The epistemological *epochē* in which one seeks understanding of the mind is a heightened state of salience and significance^37^. These instincts are implicated in imaginative cultures as part of the experience of organizing sensible affective knowledge.

Knowledge is itself a reflexive mimetic activity, this can be observed in trance and possession as forms of acquaintance that transform the knower at the moment he learns (Calasso, 2020). The impulse to believe, the desire to know, and the feeling of immanence, or salience, spur the mind toward epistemic closure. The feeling of presence, that is, the emboldening direct experience of mental contents, may be the best grounds for experiencing an attainment of truth (Otto, 1904). This knowledge by acquaintance was the foundation of Williams James' radical empiricism (James and Castell, 1948). The forms in which belief is materialized in religious and art practices vary widely because they employ sets of symbols drawn from the local epistemic niche^38^. Ritual manifests the liturgical order by engendering the feeling of immanence and canalizing metaphysical belief into local knowledge practices. These are the grounds upon which the logos is enforced (Rappaport, 1999, p. 396). In this paper, art is portrayed as that space for reflexive knowledge which serves purposes of solidifying cultural norms through belief fixation. This process relies upon the decisive authority of the felt sense of immanence. In addition to adding a vivid sense of pleasure, art conscripts bodily, affective, and imaginative processes to mediate the attainment of such sensible affective forms of knowledge.

## Author Contributions

The author confirms being the sole contributor of this work and has approved it for publication.

## Conflict of Interest

The author declares that the research was conducted in the absence of any commercial or financial relationships that could be construed as a potential conflict of interest.

## Publisher's Note

All claims expressed in this article are solely those of the authors and do not necessarily represent those of their affiliated organizations, or those of the publisher, the editors and the reviewers. Any product that may be evaluated in this article, or claim that may be made by its manufacturer, is not guaranteed or endorsed by the publisher.

## Footnotes

^1^On the relation between broader cultural projects and psychology, see Noë (2015), Asma and Gabriel (2019), and Damasio (2019).

^2^The concept of immanence has been discussed under other names, such as salience and presentness, it shares similarity with this definition of enchantment: …*a state of wonder…the temporary suspension of chronological time and bodily movement. To be enchanted, then, is to participate in a momentarily immobilizing encounter; it is to be transfixed, spellbound…Contained within this surprise state are (1) a pleasurable feeling of being charmed by the novel and as yet unprocessed encounter and (2) a more unheimlich (uncanny) feeling of being disrupted or torn out of one's default sensory-psychic-intellectual disposition*. (Bennett, 2001, p. 5). See also Bataille (1948/1989).

^3^Numinous rerefers to the non-discursive, affective, ineffable qualities of relation, whereas sacred signifies the discursive aspects of religion, which can be expressed in language, see Rappaport (1999). On basic affective mechanisms, see Panksepp (1998). On how spiritual emotions are related to basic emotions and may have arisen through domestication of affect, see Asma and Gabriel (2019).

^4^See also Austin (1962) on performative acts.

^5^Rappaport compares Logos to the ancient Egyptian term Ma'at, the Vedic term Ṛta, the Sioux Wakan-tanka, Orenda of the Iroquois, pokunt of the Shoshone, Manitou of the Algonquin, and Nauala of the Kwakiutl peoples, and the early Iranian term Asha, which all served similar functions in the respective societies. It is worthwhile to consider which terms are used now to denote ultimate orders, some possibilities are Nature and Biology, see Gabriel (2021b).

^6^See discussion of Rudolph Otto's *The Idea of the Holy* (1917) in Rappaport (1999, p. 377).

^7^On fear and anxiety see Malinowski (1922). On conflicting drives and emotions see Freud (1907). On sublimation, see Turner (1969).

^8^“(T)he older and more foundational function of mind is ‘hedonic sharpening' or, if you like, ‘biological flourishing;' not accuracy of indicative models and theories.” See Asma (in press).

^9^Charles Morgan quoted in Geertz (1983, p. 28). Also, see Cavell (1979, p. 159), “To satisfy the wish for the worlds' exhibition we must be willing to let the world as such appear. According to Heidegger this means that we must be willing for anxiety, to which alone the world as world, into which we are thrown, can manifest itself; and it is through that willingness that the possibility of one's own existence begins or ends. To satisfy the wish to act without performing, to let our actions go out of our hands, we must be willing to allow the self to exhibit itself without the self's intervention. The wish for total intelligibility is a terrible one. It means that we are willing to reveal ourselves through the self's betrayal of itself.” Though see Fried (1998) on theatricality and objecthood.

^10^The mystification inherent in the emotional response to many works of art and ritual ceremonies draws from the occult power of collective effervescence and the confrontation of multiple orders and disorders. See Rappaport (1999, p. 48 n11).

^11^Asma and Gabriel (2019). See Chapter 9.

^12^Or, in Cavell's (1979, p. 110) words, “Painting, being art, is revelation; it is revelation because it is acknowledgment; being acknowledgment, it is knowledge, of itself, and of the world”.

^13^Analogy, as Levi-Strauss claims, is also a quality of wild thought which creates order by making connections between ideas. Freud as well as the British Empiricists thought that the mind is intrinsically given to making associations, whether in primary processes and the dream-work or through laws of association.

^14^On ‘line of flight,' see Deleuze and Guattari (1987/2013). See also Tan (2018).

^15^See Kant's Critique of the Power of Judgment (1790), and Hegel's Lectures on Fine Arts given between 1818 and 1829 and collected in Hegel and Knox (1998).

^16^For Pippin (2014, p. 16), aesthetics allow for “a reconciliation of sorts between the inescapably finite, constrained, natural embodied features of human existence and the practically undeniable, meaning- and norm-responsive, reflective, self-determining features.” See also Tuan (1993).

^17^Gilles Deleuze (1986) claims that the crisis of empirical psychology to unite idealism and materialism, as seen in the work of Henri Bergson and Edmund Husserl, coincides with the rise of cinema as a method to unify movement, action, and emotion in moving images.

^18^These forms of intermediate aspects of mind are discussed in detail in Asma and Gabriel (2019).

^19^This is akin to Nietzsche's notion of the genealogy of knowledge in which our consideration of the timeliness of the idea, its changing character allows us to recognize that value and meaning are themselves relational and contextual.

^20^See Chapters 7 and 8 of Asma and Gabriel (2019).

^21^Though they are energized by the lower level emotions, ruminations and thoughts, represented in language, symbols, and directed by executive control constitute the tertiary-level. These serve as top-down regulators and directors of emotion. At this third level we arrive at uniquely human emotions that include cognitive executive functions, emotional ruminations and regulations, and conscious agency.

^22^On image grammar, see Barsalou (1999). On body-task grammar, see Barton (2012). On non-conceptual content, see Bermúdez and Cahen (2015).

^23^See Chapter 2 of Harrison (1913).

^24^Contemporary performative art forms like the rock concert and experimental theater attempt to render the audience into a type of congregation. Moreover, social media allows for a mode of contemplative participation through spectral self-presentation.

^25^Narrative formats of drama elicit emotional responses, for example, *Romance* gives narrative expression to the sexual desire and longing (i.e., LUST and SEEKING), while the typical *horror* plot is a narrative expression of terror (i.e., FEAR). *Tragedy* engages feelings of grief (i.e., separation distress system), while *mysteries* and hero stories engage tantalizing meanderings (of the SEEKING system). See Asma (in press).

^26^Film is magical and thus sacred for Stanley Cavell because it satisfies the wish for “the magical reproduction of the world by enabling us to view it unseen…to see in this way…the world itself” (Cavell, 1979, p. 101).

^27^Stanley Cavell (1979, p. 6) puts this nicely: “It is generally true that you do not really like the highest instances unless you also like the typical ones. You don't even know what the highest are instances of unless you know the typical as well”.

^28^With the ubiquity of video-recording technology, questions arise about what attempts to “capture the moment” mean: What is the psychological import of recording itself? Do individuals record events as a way of sanctifying them relative to prosaic life? Does recording entail a form of techno-sacralization?.

^29^“We are tantalized at once by our subjection to it and by its subjection to our views of it” (Cavell, 1979, p. 189).

^30^Image does not just refer to visual sensation, complex sounds and touch perception are also images. See Damasio (2012).

^31^According to Walter Freeman, “the role of trance states was particularly important for breaking down preexisting habits and beliefs. That meltdown appears to be necessary for personality changes leading to the formation of social groups by cooperative action leading to trust. Bonding is not simply a release of a neurochemical in an altered state. It is the social action of dancing and singing together that induces new forms of behavior, owing to the malleability that can come through the altered state.” (Freeman, 2000, p. 422). Bateson (1972) calls this third-level learning.

^32^This making present is called “presentness” by Michael Fried, and for Hegel art has the task of bringing the Absolute to presentness.

^33^Modern art can be considered a dramatization of how our condition implicates a crisis of meaning, political strife, theatricality, consumer fetishism, and a lack of coherence due to the surfeit of information that came along with technical mastery. Modern art thus discloses the immanence of such issues in the intention of the artist and in truths that transcend the artist's intentions. See the chapter on Heidegger's view of art as a deed of unconcealment in Pippin (2014).

^34^Penguin dictionary of Psychology quoted in Lewis (1989, p. 33).

^35^An epidemiology of possession indicates that individuals can modify their socio-political standing through their role in these rituals. See Lewis (1989).

^36^Herbert quotes Nettl (2000, p. 468) in describing music as the crucial component of ritual and link to the supernatural found in some form in all societies.

^37^Cf. Nugent (2017) on fascination as being enchanted by the nature of phenomenal consciousness.

^38^Indeed, the scientific method may be one such ritualized response to the hazard of ignorance (Foss, 2007).
